# Crystal structure of *trans*-(1,8-dibutyl-1,3,6,8,10,13-hexa­aza­cyclo­tetra­decane-κ^4^ *N* ^3^,*N* ^6^,*N* ^10^,*N* ^13^)bis­(5-methyltetra­zolato-κ*N*)nickel(II) from synchrotron data

**DOI:** 10.1107/S2056989015000651

**Published:** 2015-01-17

**Authors:** Dae-Woong Kim, Jong Won Shin, Jin Hong Kim, Dohyun Moon

**Affiliations:** a Beamline Department, Pohang Accelerator Laboratory, 80 Jigokro-127-beongil, Nam-Gu Pohang, Gyeongbuk 790-784, Republic of Korea

**Keywords:** crystal structure, aza­macrocyclic ligand, Jahn–Teller distortion, tetra­zole derivatives, synchrotron data

## Abstract

The structure of the title compound, [Ni(C_2_H_3_N_4_)_2_(C_16_H_38_N_6_)], has been characterized from synchrotron radiation. The asymmetric unit consists of one half of the Ni^II^ complex mol­ecule, which is related to the other half-mol­ecule by an inversion center. The Ni^II^ ion is coordinated by four secondary N atoms of the macrocyclic ligand in a square-planar fashion in the equatorial plane and by two N atoms of the 5-methyltetra­zolate anions in axial positions, resulting in a tetra­gonally distorted octa­hedral geometry. The average equatorial Ni—N bond length [2.060 (8) Å] is shorter than the axial Ni—N bond length [2.2183 (11) Å]. An intra­molecular N—H⋯N hydrogen bond between the secondary amine N atom of the macrocyclic ligand and the non-coordinating N atom of the 5-methyltetra­zolate ion stabilizes the mol­ecular structure. Moreover, an inter­molecular N—H⋯N hydrogen bond between the macrocyclic ligand and 5-methyltetra­zolate group gives rise to a supra­molecular sheet structure parallel to the *bc* plane.

## Abstract 2

The Ni^II^ ion in the title compound shows a slightly distorted octa­hedral coordination geometry with four N atoms of the aza­macrocylic ligand and two N atoms of the 5-methyl-1*H*-tetra­zolate ions. In the crystal, mol­ecules are connected by an N—H⋯N hydrogen bond, forming a supra­molecular sheet structure.

## Chemical context   

Coordination compounds with macrocyclic ligands have been studied widely in chemistry, metalloenzymes and materials science (Lehn, 1995 ▸). In particular, Ni^II^ macrocyclic complexes having vacant sites in the axial positions are good building blocks for assembling supra­molecular frameworks (Min & Suh, 2001 ▸), with potential applications in gas adsorption/desorption (Lee & Suh, 2004 ▸), carbon dioxide reduction (Froehlich & Kubiak, 2012 ▸) and chiral separation (Ryoo *et al.*, 2010 ▸). For example, Ni^II^ complexes with tetra-aza­macrocyclic ligands have been studied as catalysts for water oxidation at neutral pH (Zhang *et al.*, 2014 ▸) and their magnetic properties have been investigated with various auxiliary anionic moieties such as azide, dicyanamide and ferricyanide (Yuan *et al.*, 2011 ▸). Moreover, tetra­zole derivatives are versatile anions which can easily bridge to transition metal ions, thus allowing the assembly of multi-dimensional compounds (Zhao *et al.*, 2008 ▸). 
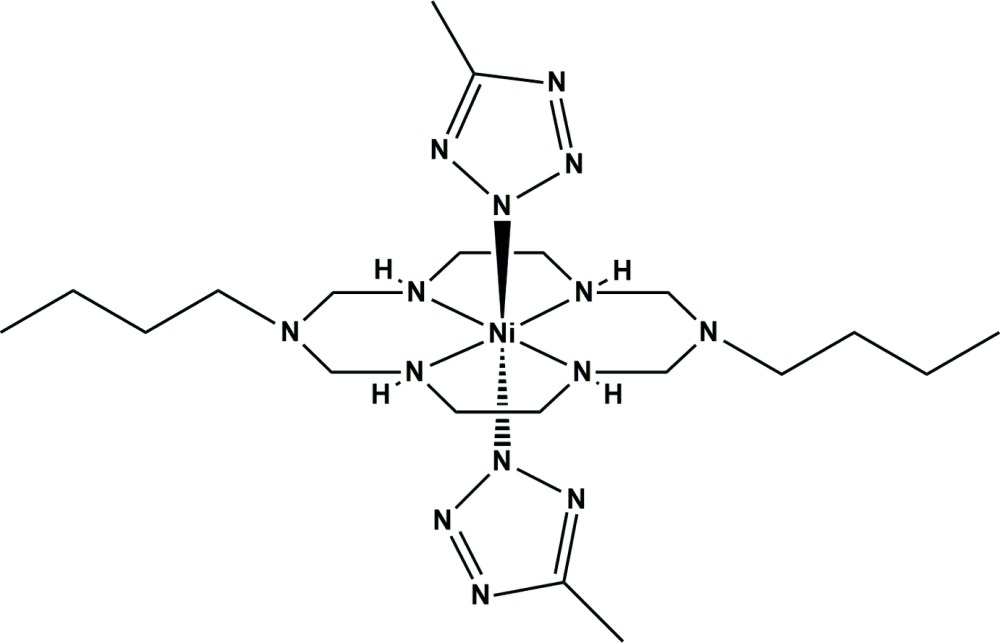


Here, we report the synthesis and crystal structure of an Ni^II^ aza­macrocyclic complex with two tetra­zole derivatives, *trans*-(1,8-dibutyl-1,3,6,8,10,13-hexa­aza­cyclo­tetra­decane-*κ* ^4^ *N* ^3^,*N* ^6^,*N* ^10^,*N* ^13^)bis­(5-methyltetra­zolato-*κN*)nickel(II), (I).

## Structural commentary   

**Figure 1:**
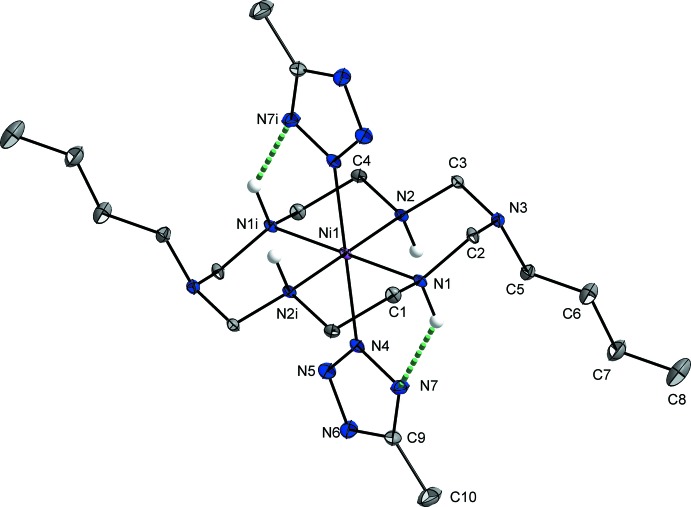
View of the mol­ecular structure of the title compound, showing the atom-labelling scheme, with displacement ellipsoids drawn at the 50% probability level. H atoms bonded to C atoms have been omitted for clarity. Intra­molecular N—H⋯N hydrogen bonds are shown as green dashed lines. [Symmetry code: (i) −*x* + 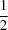, −*y* + 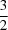, −*z* + 1.]

In the title compound, the coordination environment around the Ni^II^ ion, in which the Ni^II^ ion lies on an inversion center, has a tetra­gonally distorted octa­hedral geometry. The Ni^II^ ion is bonded to four secondary N atoms of the aza­macrocyclic ligand in a square-planar fashion in the equatorial plane, and to two N atoms from the 5-methyltetra­zolate anions at the axial positions, as shown in Fig. 1 ▸. The average Ni—N_eq_ bond length and the Ni—N_ax_ length are 2.060 (8) and 2.2183 (11) Å, respectively. The axial bond lengths are much longer than the equatorial bond lengths, which can be attributed to a rather large Jahn–Teller distortion of the Ni^II^ ion and/or ring contraction of the aza­macrocyclic ligand (Halcrow, 2013 ▸). The six-membered chelate rings adopt chair conformations and the five-membered chelate rings assume *gauche* conformations (Min & Suh, 2001 ▸). The N—N bond lengths in the 5-methyl­tetra­zolate ion range from 1.3182 (15) to 1.3543 (16) Å, indicating that the tetra­zolate ring is fully delocalized. An intra­molecular N—H⋯N hydrogen bond between the secondary amine group of the macrocyclic ligand and the N atom of the 5-methyltetra­zolate ion stabilizes the mol­ecular structure (Fig. 1 ▸ and Table 1 ▸).

## Supra­molecular features   

**Figure 2:**
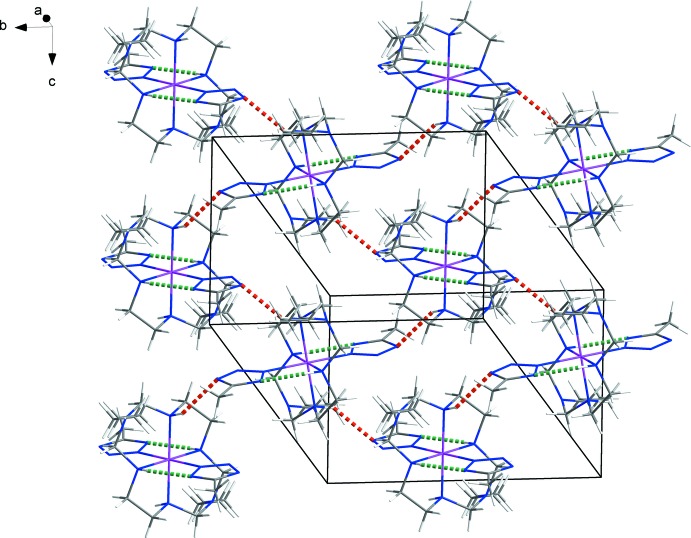
View of the crystal packing of the title compound, with N—H⋯N hydrogen bonds drawn as green (intra­molecular) and red (inter­molecular) dashed lines.

The packing in the structure involves an inter­molecular N—H⋯N hydrogen bond between the secondary amine group of the macrocyclic ligand and the non-coordinating N atom of the 5-methyltetra­zolate ion (Table 1 ▸), which forms a rigid supra­molecular sheet structure parallel to the *bc* plane (Fig. 2 ▸).

## Database survey   

A search of the Cambridge Structural Database (Version 5.35, May 2014 with 3 updates; Groom & Allen, 2014 ▸) indicated that 71 Ni^II^ aza­macrocyclic complexes with alkyl pendant groups have been reported. These complexes with various alkyl pendant groups were investigated as good building blocks for supra­molecular chemistry and also studied for their magnetic properties and gas sorption abilities due to the anions such as cyanido groups and carb­oxy­lic acid derivatives (Hyun *et al.*, 2013 ▸; Shen *et al.*, 2012 ▸). No corresponding Ni^II^ aza­macrocyclic complex with a butyl pendant group and tetra­zole derivatives has been reported, and the title compound was newly synthesized for this research.

## Synthesis and crystallization   

The title compound (I) was prepared as follows. The starting complex, [Ni(C_16_H_38_N_6_)(ClO_4_)_2_], was prepared by a slight modification of the reported method (Jung *et al.*, 1989 ▸). To an MeCN (10 mL) solution of [Ni(C_16_H_38_N_6_)(ClO_4_)_2_] (0.10 g, 0.17 mmol) was slowly added an MeCN solution (5 mL) containing 5-methyl-1*H*-tetra­zole (0.029 g, 0.34 mmol) and excess tri­ethyl­amine (0.04 g, 0.40 mmol) at room temperature. The color of the solution turned from yellow to pale pink and a pale-pink precipitate was formed, which was filtered off, washed with MeCN, and diethyl ether, and dried in air. Single crystals of the title compound were obtained by layering of the MeCN solution of 5-methyl-1*H*-tetra­zole on the MeCN solution of [Ni(C_16_H_38_N_6_)(ClO_4_)_2_] for several days. Yield: 0.057 g (62%). FT–IR (ATR, cm^−1^): 3215, 2954, 1590, 1488, 1457, 1376, 1237, 1019, 933.

**Safety note:** Although we have experienced no problem with the compounds reported in this study, perchlorate salts of metal complexes are often explosive and should be handled with great caution.

## Refinement   

Crystal data, data collection and structure refinement details are summarized in Table 2 ▸. All H atoms were placed in geometrically idealized positions and constrained to ride on their parent atoms, with C—H = 0.98–0.99 Å and N—H = 1.00 Å, and with *U*_iso_(H) values of 1.2 or 1.5*U*_eq_ of the parent atoms.

## Supplementary Material

Crystal structure: contains datablock(s) I. DOI: 10.1107/S2056989015000651/is5389sup1.cif

Structure factors: contains datablock(s) I. DOI: 10.1107/S2056989015000651/is5389Isup2.hkl

CCDC reference: 1043241

Additional supporting information:  crystallographic information; 3D view; checkCIF report

## Figures and Tables

**Table 1:** Hydrogen-bond geometry (, )

*D*H*A*	*D*H	H*A*	*D* *A*	*D*H*A*
N1H1N7	1.00	2.07	2.8508(16)	133
N2H2N6^i^	1.00	2.35	3.1403(16)	135

Symmetry code: (i) 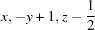.

**Table 2:** Experimental details

Crystal data	
Chemical formula	[Ni(C_2_H_3_N_4_)_2_(C_16_H_38_N_6_)]
*M*_r_	539.40
Crystal system, space group	Monoclinic, *C*2/*c*
Temperature (K)	100
*a*, *b*, *c* ()	24.040(5), 12.923(3), 8.7170(17)
()	98.94(3)
*V* (^3^)	2675.1(9)
*Z*	4
Radiation type	Synchrotron, = 0.62998
(mm^1^)	0.55
Crystal size (mm)	0.05 0.04 0.04

Data collection	
Diffractometer	ADSC Q210 CCD area detector
Absorption correction	Empirical (using intensity measurements) (*HKL3000sm *SCALEPACK**; Otwinowski Minor, 1997 ▸)
*T*_min_, *T*_max_	0.973, 0.978
No. of measured, independent and observed [*I* > 2(*I*)] reflections	12808, 3761, 3150
*R*_int_	0.042
(sin /)_max_ (^1^)	0.696

Refinement	
*R*[*F* ^2^ > 2(*F* ^2^)], *wR*(*F* ^2^), *S*	0.032, 0.090, 1.08
No. of reflections	3761
No. of parameters	162
H-atom treatment	H-atom parameters constrained
_max_, _min_ (e ^3^)	0.32, 0.79

Computer programs: *PAL ADSC Quantum-210 ADX* (Arvai Nielsen, 1983 ▸), *HKL3000sm* (Otwinowski Minor, 1997 ▸), *SHELXT2014/5* (Sheldrick, 2015*a* ▸), *SHELXL2014/7* (Sheldrick, 2008 ▸, 2015*b* ▸), *DIAMOND4* (Putz Brandenburg, 2014 ▸) and *publCIF* (Westrip, 2010 ▸).

## supplementary crystallographic information

### Crystal data

**Table:**

[Ni(C_2_H_3_N_4_)_2_(C_16_H_38_N_6_)]	*F*(000) = 1160
*M**_r_* = 539.40	*D*_x_ = 1.339 Mg m^−^^3^
Monoclinic, *C*2/*c*	Synchrotron radiation, λ = 0.62998 Å
*a* = 24.040 (5) Å	Cell parameters from 25946 reflections
*b* = 12.923 (3) Å	θ = 0.4–33.6°
*c* = 8.7170 (17) Å	µ = 0.55 mm^−^^1^
β = 98.94 (3)°	*T* = 100 K
*V* = 2675.1 (9) Å^3^	Block, pink
*Z* = 4	0.05 × 0.04 × 0.04 mm

### Data collection

**Table:**

ADSC Q210 CCD area-detector diffractometer	3150 reflections with *I* > 2σ(*I*)
Radiation source: PLSII 2D bending magent	*R*_int_ = 0.042
ω scan	θ_max_ = 26.0°, θ_min_ = 1.6°
Absorption correction: empirical (using intensity measurements) (*HKL3000sm *SCALEPACK**; Otwinowski & Minor, 1997)	*h* = −33→33
*T*_min_ = 0.973, *T*_max_ = 0.978	*k* = −17→17
12808 measured reflections	*l* = −12→12
3761 independent reflections	

### Refinement

**Table:**

Refinement on *F*^2^	0 restraints
Least-squares matrix: full	Hydrogen site location: inferred from neighbouring sites
*R*[*F*^2^ > 2σ(*F*^2^)] = 0.032	H-atom parameters constrained
*wR*(*F*^2^) = 0.090	*w* = 1/[σ^2^(*F*_o_^2^) + (0.0565*P*)^2^ + 0.106*P*] where *P* = (*F*_o_^2^ + 2*F*_c_^2^)/3
*S* = 1.08	(Δ/σ)_max_ < 0.001
3761 reflections	Δρ_max_ = 0.32 e Å^−^^3^
162 parameters	Δρ_min_ = −0.79 e Å^−^^3^

### Special details

**Table:**

Geometry. All e.s.d.'s (except the e.s.d. in the dihedral angle between two l.s. planes) are estimated using the full covariance matrix. The cell e.s.d.'s are taken into account individually in the estimation of e.s.d.'s in distances, angles and torsion angles; correlations between e.s.d.'s in cell parameters are only used when they are defined by crystal symmetry. An approximate (isotropic) treatment of cell e.s.d.'s is used for estimating e.s.d.'s involving l.s. planes.

### Fractional atomic coordinates and isotropic or equivalent isotropic displacement parameters (Å^2^)

**Table:**

	*x*	*y*	*z*	*U*_iso_*/*U*_eq_	
Ni1	0.2500	0.7500	0.5000	0.00596 (8)	
N1	0.31782 (5)	0.83411 (8)	0.60560 (11)	0.0073 (2)	
H1	0.3488	0.7841	0.6424	0.009*	
N2	0.28136 (5)	0.73476 (8)	0.29352 (12)	0.0076 (2)	
H2	0.3095	0.6773	0.3067	0.009*	
N3	0.35616 (5)	0.86428 (8)	0.36376 (12)	0.0095 (2)	
C1	0.29951 (5)	0.88036 (9)	0.74420 (13)	0.0094 (2)	
H1A	0.2752	0.9411	0.7137	0.011*	
H1B	0.3327	0.9038	0.8177	0.011*	
C2	0.34005 (6)	0.90993 (9)	0.50278 (14)	0.0097 (2)	
H2A	0.3110	0.9634	0.4716	0.012*	
H2B	0.3733	0.9448	0.5621	0.012*	
C3	0.31026 (6)	0.82902 (9)	0.24815 (14)	0.0096 (2)	
H3A	0.3251	0.8147	0.1505	0.011*	
H3B	0.2823	0.8854	0.2270	0.011*	
C4	0.23296 (6)	0.70064 (10)	0.17841 (14)	0.0095 (2)	
H4A	0.2464	0.6706	0.0864	0.011*	
H4B	0.2083	0.7603	0.1441	0.011*	
C5	0.40350 (6)	0.79167 (10)	0.39114 (15)	0.0119 (2)	
H5A	0.4070	0.7560	0.2926	0.014*	
H5B	0.3955	0.7386	0.4666	0.014*	
C6	0.45926 (6)	0.84385 (12)	0.45282 (18)	0.0188 (3)	
H6A	0.4686	0.8939	0.3747	0.023*	
H6B	0.4553	0.8829	0.5483	0.023*	
C7	0.50699 (7)	0.76635 (13)	0.4891 (2)	0.0237 (3)	
H7A	0.5101	0.7263	0.3940	0.028*	
H7B	0.4977	0.7172	0.5685	0.028*	
C8	0.56368 (7)	0.81669 (16)	0.5480 (2)	0.0334 (4)	
H8A	0.5737	0.8641	0.4688	0.050*	
H8B	0.5926	0.7630	0.5696	0.050*	
H8C	0.5612	0.8554	0.6434	0.050*	
N4	0.30074 (5)	0.61260 (8)	0.58628 (12)	0.0100 (2)	
N5	0.29007 (5)	0.51298 (9)	0.56721 (14)	0.0156 (2)	
N6	0.33266 (5)	0.45876 (9)	0.64836 (16)	0.0185 (3)	
N7	0.35088 (5)	0.62585 (9)	0.67914 (13)	0.0146 (2)	
C9	0.36869 (6)	0.52976 (10)	0.71493 (17)	0.0157 (3)	
C10	0.42228 (7)	0.50538 (13)	0.8200 (2)	0.0298 (4)	
H10A	0.4139	0.4869	0.9230	0.045*	
H10B	0.4470	0.5661	0.8285	0.045*	
H10C	0.4411	0.4472	0.7773	0.045*	

### Atomic displacement parameters (Å^2^)

**Table:**

	*U*^11^	*U*^22^	*U*^33^	*U*^12^	*U*^13^	*U*^23^
Ni1	0.00867 (12)	0.00459 (12)	0.00474 (11)	−0.00101 (8)	0.00141 (8)	−0.00027 (7)
N1	0.0102 (5)	0.0052 (5)	0.0069 (4)	−0.0004 (4)	0.0021 (4)	−0.0002 (4)
N2	0.0092 (5)	0.0077 (5)	0.0061 (4)	−0.0007 (4)	0.0016 (4)	−0.0005 (4)
N3	0.0085 (5)	0.0105 (5)	0.0097 (5)	−0.0006 (4)	0.0024 (4)	0.0003 (4)
C1	0.0120 (6)	0.0083 (5)	0.0080 (5)	−0.0012 (4)	0.0016 (4)	−0.0031 (4)
C2	0.0114 (6)	0.0068 (5)	0.0115 (5)	−0.0016 (4)	0.0032 (4)	0.0001 (4)
C3	0.0112 (6)	0.0095 (6)	0.0084 (5)	−0.0012 (4)	0.0026 (4)	0.0022 (4)
C4	0.0112 (6)	0.0109 (6)	0.0062 (5)	0.0000 (5)	0.0012 (4)	−0.0009 (4)
C5	0.0099 (6)	0.0126 (6)	0.0136 (6)	0.0010 (5)	0.0029 (5)	0.0006 (5)
C6	0.0117 (7)	0.0189 (7)	0.0250 (7)	−0.0002 (5)	0.0008 (5)	−0.0032 (6)
C7	0.0128 (7)	0.0257 (8)	0.0316 (8)	0.0019 (6)	−0.0001 (6)	0.0016 (6)
C8	0.0139 (8)	0.0426 (10)	0.0409 (10)	0.0023 (7)	−0.0038 (7)	−0.0053 (8)
N4	0.0123 (5)	0.0071 (5)	0.0108 (5)	−0.0002 (4)	0.0024 (4)	0.0009 (4)
N5	0.0149 (6)	0.0081 (5)	0.0233 (6)	0.0003 (4)	0.0017 (5)	0.0026 (4)
N6	0.0129 (6)	0.0099 (5)	0.0323 (7)	0.0017 (4)	0.0025 (5)	0.0051 (5)
N7	0.0134 (6)	0.0110 (5)	0.0180 (5)	0.0013 (4)	−0.0016 (4)	0.0027 (4)
C9	0.0127 (6)	0.0113 (6)	0.0233 (7)	0.0019 (5)	0.0028 (5)	0.0060 (5)
C10	0.0194 (8)	0.0199 (7)	0.0460 (10)	0.0024 (6)	−0.0079 (7)	0.0092 (7)

### Geometric parameters (Å, º)

**Table:**

Ni1—N1	2.0543 (12)	C5—C6	1.522 (2)
Ni1—N2	2.0661 (11)	C5—H5A	0.9900
Ni1—N4	2.2183 (11)	C5—H5B	0.9900
N1—C1	1.4752 (15)	C6—C7	1.519 (2)
N1—C2	1.4826 (15)	C6—H6A	0.9900
N1—H1	1.0000	C6—H6B	0.9900
N2—C4	1.4807 (17)	C7—C8	1.525 (2)
N2—C3	1.4860 (16)	C7—H7A	0.9900
N2—H2	1.0000	C7—H7B	0.9900
N3—C3	1.4474 (17)	C8—H8A	0.9800
N3—C2	1.4535 (16)	C8—H8B	0.9800
N3—C5	1.4657 (17)	C8—H8C	0.9800
C1—C4^i^	1.5244 (17)	N4—N5	1.3182 (15)
C1—H1A	0.9900	N4—N7	1.3543 (16)
C1—H1B	0.9900	N5—N6	1.3469 (17)
C2—H2A	0.9900	N6—C9	1.3314 (19)
C2—H2B	0.9900	N7—C9	1.3347 (17)
C3—H3A	0.9900	C9—C10	1.494 (2)
C3—H3B	0.9900	C10—H10A	0.9800
C4—C1^i^	1.5244 (17)	C10—H10B	0.9800
C4—H4A	0.9900	C10—H10C	0.9800
C4—H4B	0.9900		
			
N1^i^—Ni1—N1	180.0	H3A—C3—H3B	107.6
N1^i^—Ni1—N2	86.04 (4)	N2—C4—C1^i^	107.88 (10)
N1—Ni1—N2	93.96 (4)	N2—C4—H4A	110.1
N1^i^—Ni1—N2^i^	93.96 (4)	C1^i^—C4—H4A	110.1
N1—Ni1—N2^i^	86.04 (4)	N2—C4—H4B	110.1
N2—Ni1—N2^i^	180.0	C1^i^—C4—H4B	110.1
N1^i^—Ni1—N4^i^	85.15 (4)	H4A—C4—H4B	108.4
N1—Ni1—N4^i^	94.86 (4)	N3—C5—C6	113.12 (11)
N2—Ni1—N4^i^	92.11 (4)	N3—C5—H5A	109.0
N2^i^—Ni1—N4^i^	87.89 (4)	C6—C5—H5A	109.0
N1^i^—Ni1—N4	94.86 (4)	N3—C5—H5B	109.0
N1—Ni1—N4	85.14 (4)	C6—C5—H5B	109.0
N2—Ni1—N4	87.89 (4)	H5A—C5—H5B	107.8
N2^i^—Ni1—N4	92.11 (4)	C7—C6—C5	112.13 (12)
N4^i^—Ni1—N4	180.0	C7—C6—H6A	109.2
C1—N1—C2	114.13 (10)	C5—C6—H6A	109.2
C1—N1—Ni1	105.32 (8)	C7—C6—H6B	109.2
C2—N1—Ni1	114.48 (8)	C5—C6—H6B	109.2
C1—N1—H1	107.5	H6A—C6—H6B	107.9
C2—N1—H1	107.5	C6—C7—C8	113.29 (14)
Ni1—N1—H1	107.5	C6—C7—H7A	108.9
C4—N2—C3	114.49 (10)	C8—C7—H7A	108.9
C4—N2—Ni1	105.31 (8)	C6—C7—H7B	108.9
C3—N2—Ni1	113.75 (7)	C8—C7—H7B	108.9
C4—N2—H2	107.7	H7A—C7—H7B	107.7
C3—N2—H2	107.7	C7—C8—H8A	109.5
Ni1—N2—H2	107.7	C7—C8—H8B	109.5
C3—N3—C2	115.80 (10)	H8A—C8—H8B	109.5
C3—N3—C5	113.60 (10)	C7—C8—H8C	109.5
C2—N3—C5	115.15 (10)	H8A—C8—H8C	109.5
N1—C1—C4^i^	108.83 (10)	H8B—C8—H8C	109.5
N1—C1—H1A	109.9	N5—N4—N7	109.66 (11)
C4^i^—C1—H1A	109.9	N5—N4—Ni1	130.78 (9)
N1—C1—H1B	109.9	N7—N4—Ni1	119.52 (8)
C4^i^—C1—H1B	109.9	N4—N5—N6	108.96 (11)
H1A—C1—H1B	108.3	C9—N6—N5	105.08 (11)
N3—C2—N1	113.80 (10)	C9—N7—N4	104.21 (11)
N3—C2—H2A	108.8	N6—C9—N7	112.09 (13)
N1—C2—H2A	108.8	N6—C9—C10	124.23 (13)
N3—C2—H2B	108.8	N7—C9—C10	123.67 (13)
N1—C2—H2B	108.8	C9—C10—H10A	109.5
H2A—C2—H2B	107.7	C9—C10—H10B	109.5
N3—C3—N2	114.21 (10)	H10A—C10—H10B	109.5
N3—C3—H3A	108.7	C9—C10—H10C	109.5
N2—C3—H3A	108.7	H10A—C10—H10C	109.5
N3—C3—H3B	108.7	H10B—C10—H10C	109.5
N2—C3—H3B	108.7		
			
C2—N1—C1—C4^i^	168.66 (10)	C2—N3—C5—C6	−68.36 (14)
Ni1—N1—C1—C4^i^	42.26 (11)	N3—C5—C6—C7	176.68 (12)
C3—N3—C2—N1	70.86 (14)	C5—C6—C7—C8	178.80 (14)
C5—N3—C2—N1	−65.05 (14)	N7—N4—N5—N6	0.63 (15)
C1—N1—C2—N3	−177.96 (10)	Ni1—N4—N5—N6	−177.06 (9)
Ni1—N1—C2—N3	−56.49 (12)	N4—N5—N6—C9	−0.27 (16)
C2—N3—C3—N2	−71.26 (14)	N5—N4—N7—C9	−0.72 (15)
C5—N3—C3—N2	65.32 (14)	Ni1—N4—N7—C9	177.28 (9)
C4—N2—C3—N3	177.73 (10)	N5—N6—C9—N7	−0.20 (17)
Ni1—N2—C3—N3	56.58 (12)	N5—N6—C9—C10	178.68 (15)
C3—N2—C4—C1^i^	−167.67 (10)	N4—N7—C9—N6	0.56 (16)
Ni1—N2—C4—C1^i^	−41.97 (10)	N4—N7—C9—C10	−178.32 (14)
C3—N3—C5—C6	154.78 (11)		

Symmetry code: (i) −*x*+1/2, −*y*+3/2, −*z*+1.

### Hydrogen-bond geometry (Å, º)

**Table:**

*D*—H···*A*	*D*—H	H···*A*	*D*···*A*	*D*—H···*A*
N1—H1···N7	1.00	2.07	2.8508 (16)	133
N2—H2···N6^ii^	1.00	2.35	3.1403 (16)	135

Symmetry code: (ii) *x*, −*y*+1, *z*−1/2.
